# Supplementary material for: Self-triggered thermoelectric nanoheterojunction for cancer catalytic and immunotherapy
Source: Nat Commun. 2023 Aug 23;14:5140. doi: 10.1038/s41467-023-40954-y (PMC10447553; doi:10.1038/s41467-023-40954-y)
Supplement: Supplementary file 1 — Supplementary Information [file 41467_2023_40954_MOESM1_ESM.pdf]

Supplementary Information

Self-Triggered Thermoelectric Nano-Heterojunction for Cancer Catalytic and Immuno-Therapy

Xue Yuan[1, †], Yong Kang[1, †], Jinrui Dong[1, †], Ruiyan Li[1], Jiamin Ye[1], Yueyue Fan[1], Jingwen Han[1], Junhui Yu[1], Guangjian Ni[1], Xiaoyuan Ji[1,2]*, Dong Ming[1]

[1]Academy of Medical Engineering and Translational Medicine, Medical College, Tianjin University, Tianjin 300072, China.

[2] Medical College, Linyi University, Linyi 276000, China

† These authors contributed equally to this work.

*****Corresponding author. Email: jixiaoyuan@tju.edu.cn

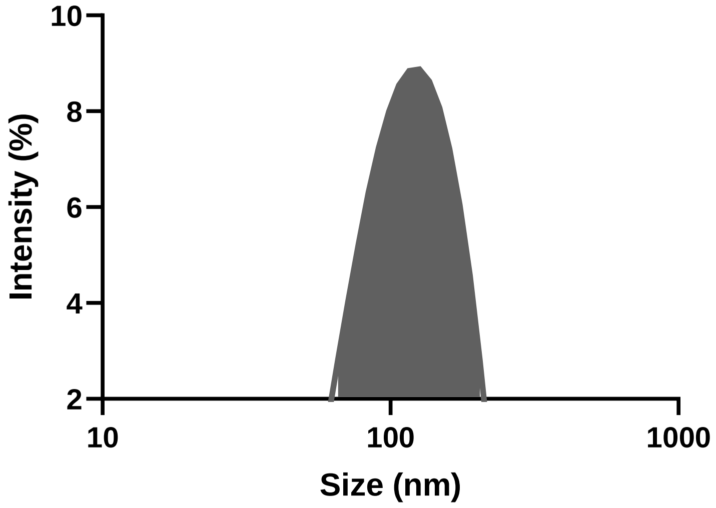

Supplementary Figure 1. Size distribution of BST NSs.

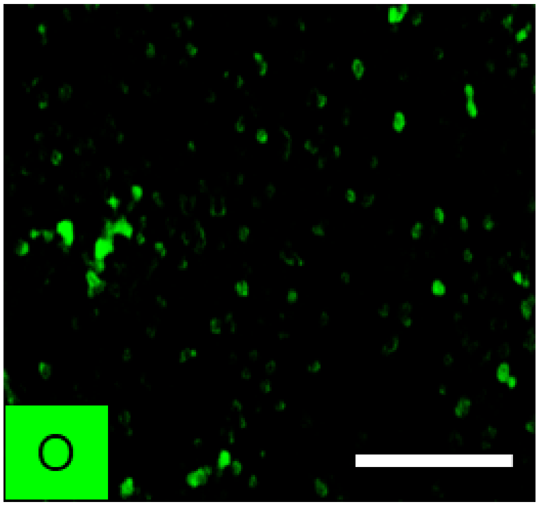

Supplementary Figure 2. EDS mapping images of O in CaO_2 NPs. Scale bar = 100 nm. Three times each experiment was repeated independently with similar results.

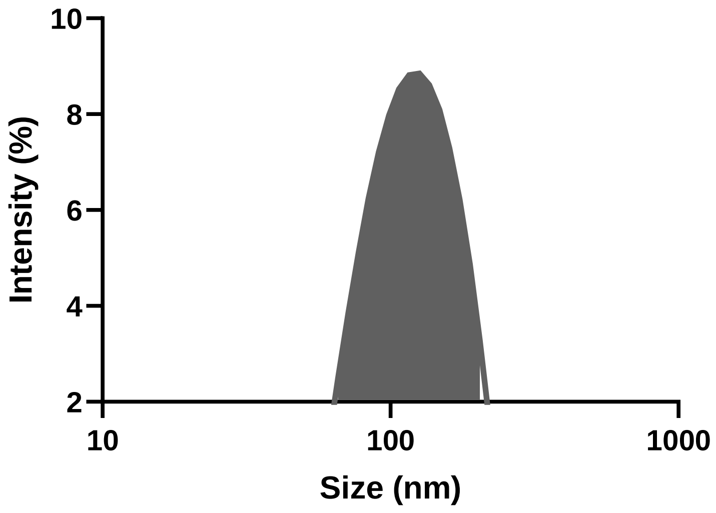

Supplementary Figure 3. Size distribution of BST/CaO_2 NSs.

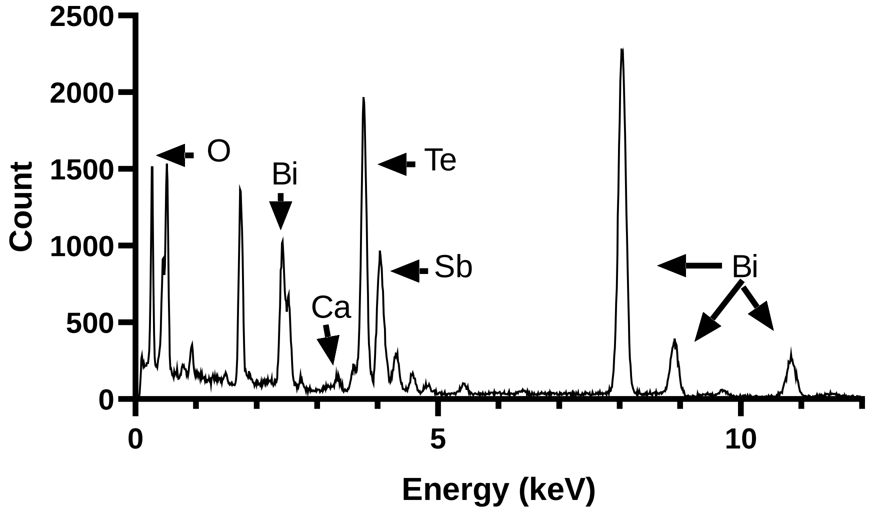

Supplementary Figure 4. EDS analysis of BST/CaO_2 NSs.

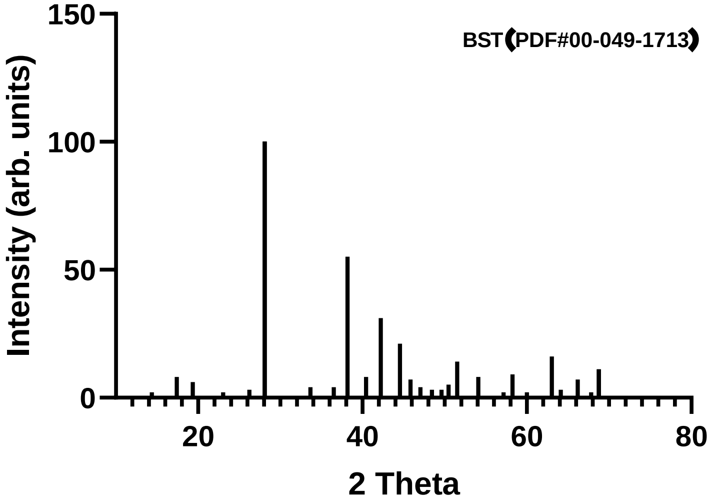

Supplementary Figure 5. Standard XRD card of BST.

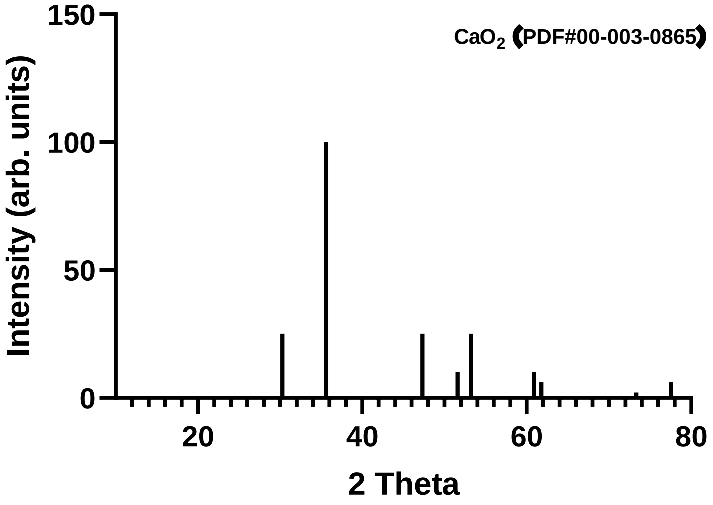

Supplementary Figure 6. Standard XRD card of CaO_2.

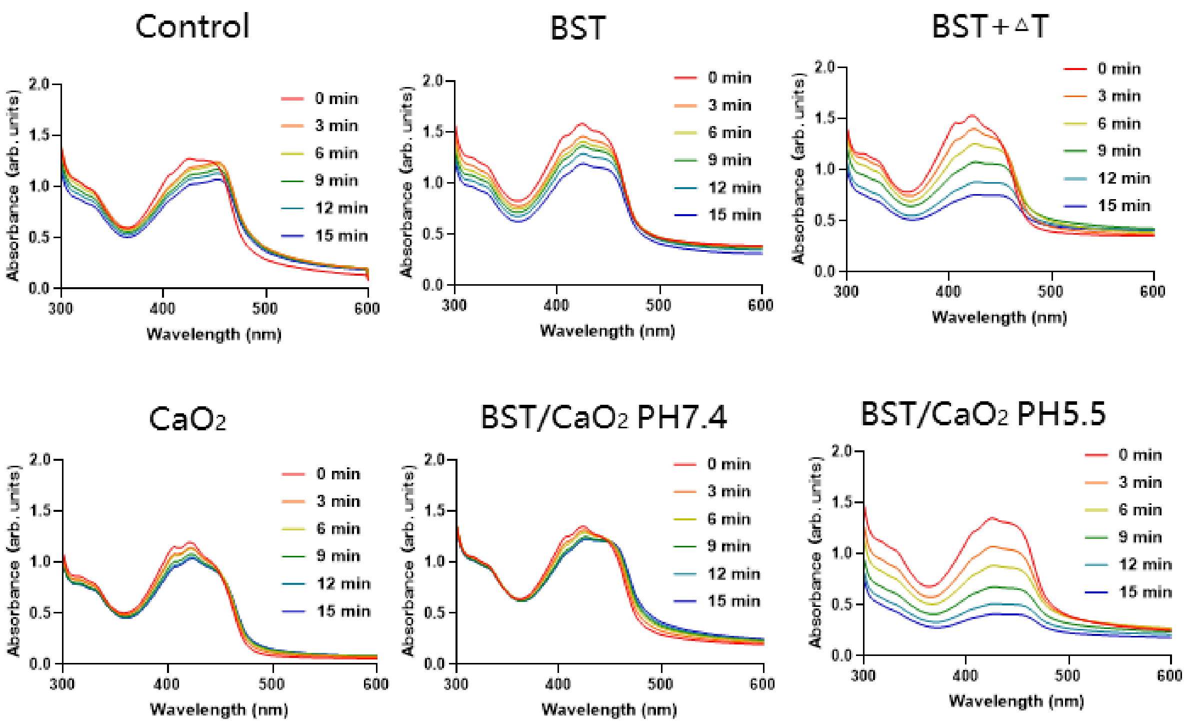

Supplementary Figure 7. Degradation of DPBF by different treatments.

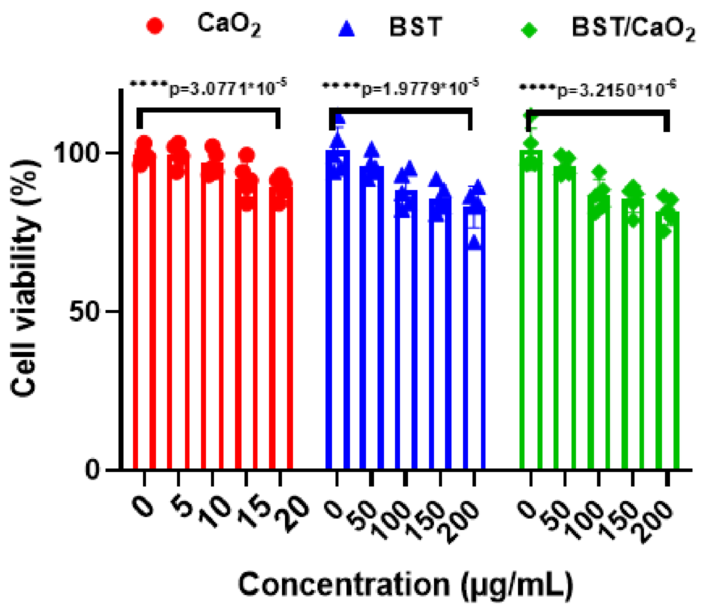

Supplementary Figure 8. The cell viability of HEK293 cells under different treatments. Data are presented as the mean ± s.d. (n = 5 biologically independent cells). Statistical differences were analyzed by Student's two-sided t test.

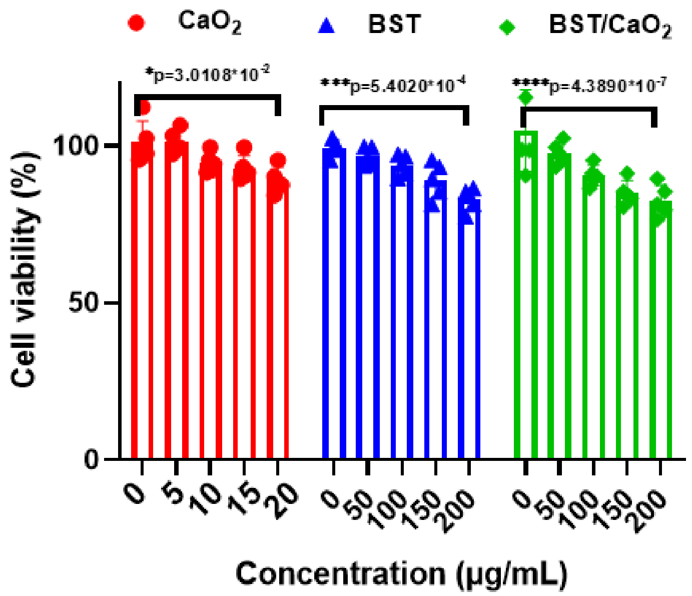

Supplementary Figure 9. The cell viability of HL-7702 cells under different treatments. Data are presented as the mean ± s.d. (n = 5 biologically independent cells). Statistical differences were analyzed by Student's two-sided t test.

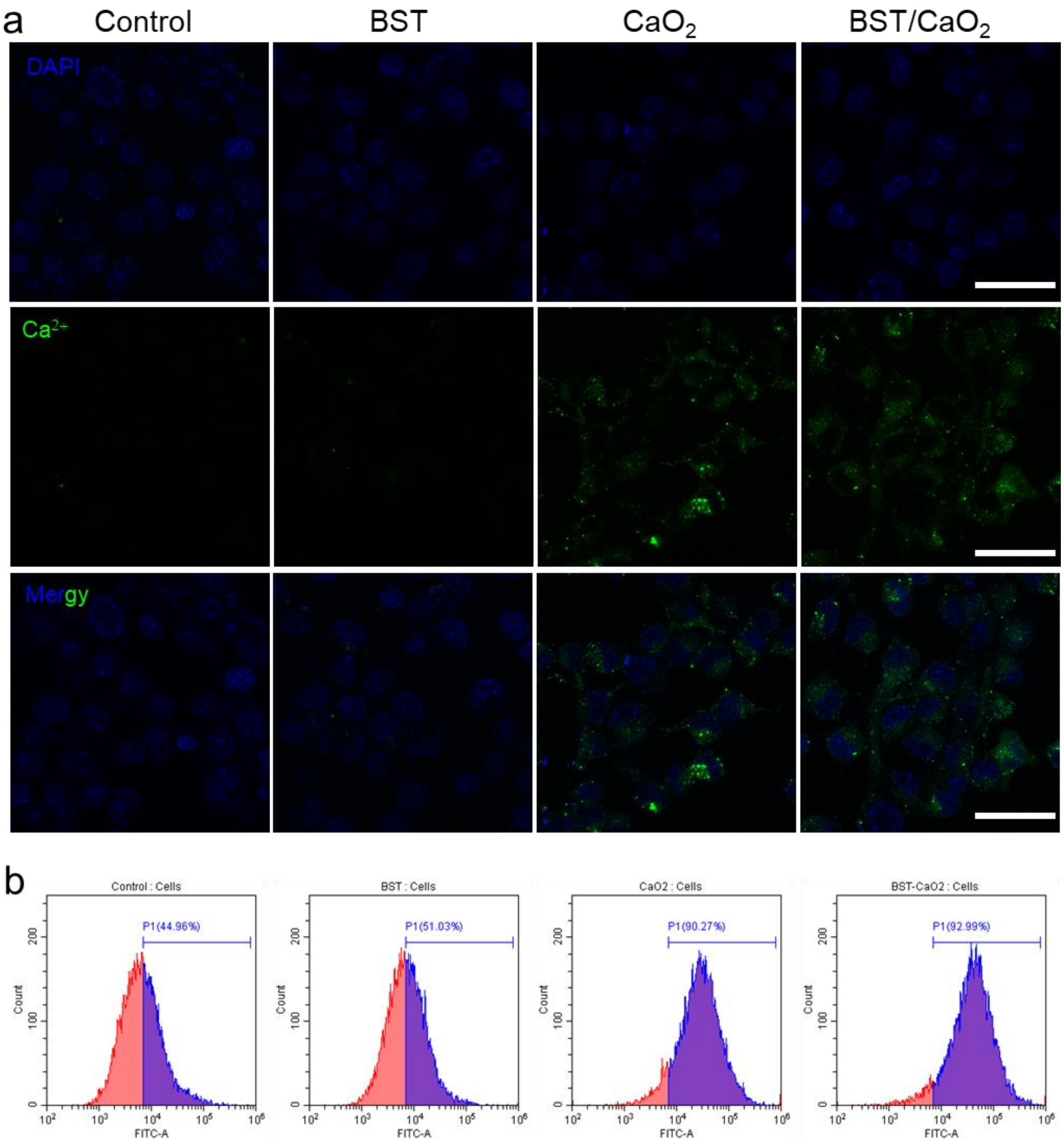

Supplementary Figure 10. Analysis of intracellular Ca^{2+} concentrations of different treatments. a Confocal laser scanning microscopy (CLSM) images and b flow cytometer (FCM) analysis of intracellular Ca^{2+} concentrations of different treatments. Scale bar = 10 μm. Three times each experiment was repeated independently with similar results.

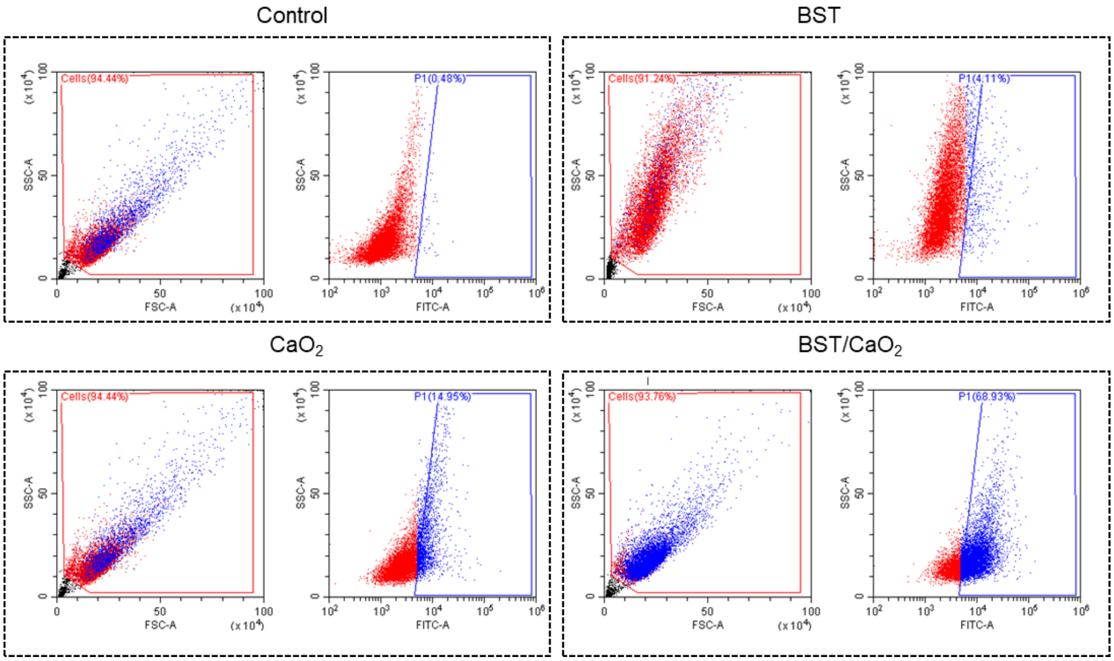

Supplementary Figure 11. Intracellular ROS generation in CT26 cells after treatment under different conditions and detection by FCM. These gating panels correspond to FACS data panels in the Fig. 5c.

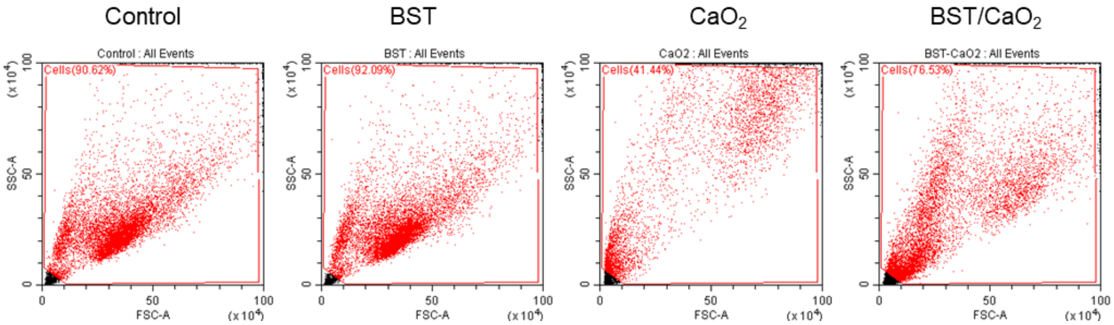

Supplementary Figure 12. FCM images of CT26 cells after treatment under different conditions. These gating panels correspond to FACS data panels in the Fig. 5h.

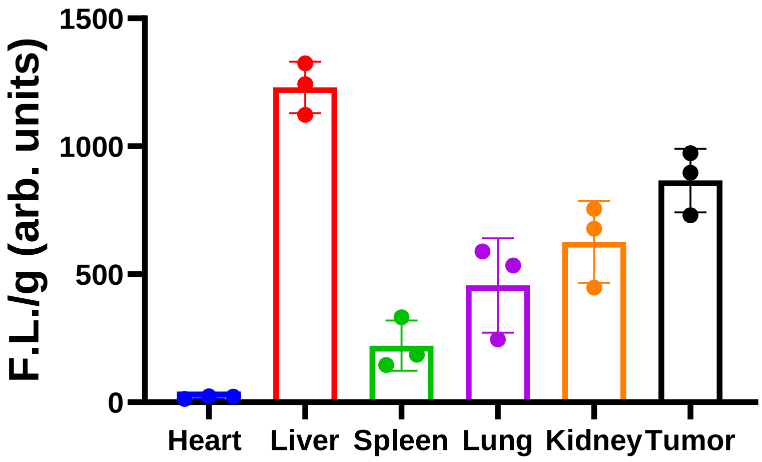

Supplementary Figure 13. Semiquantitative analysis of the biodistribution of Cy5.5-labeled BST/CaO_2 NSs in CT26 xenograft tumor-bearing mice. Data are presented as the mean ± s.d. (n = 3 biologically independent mice).

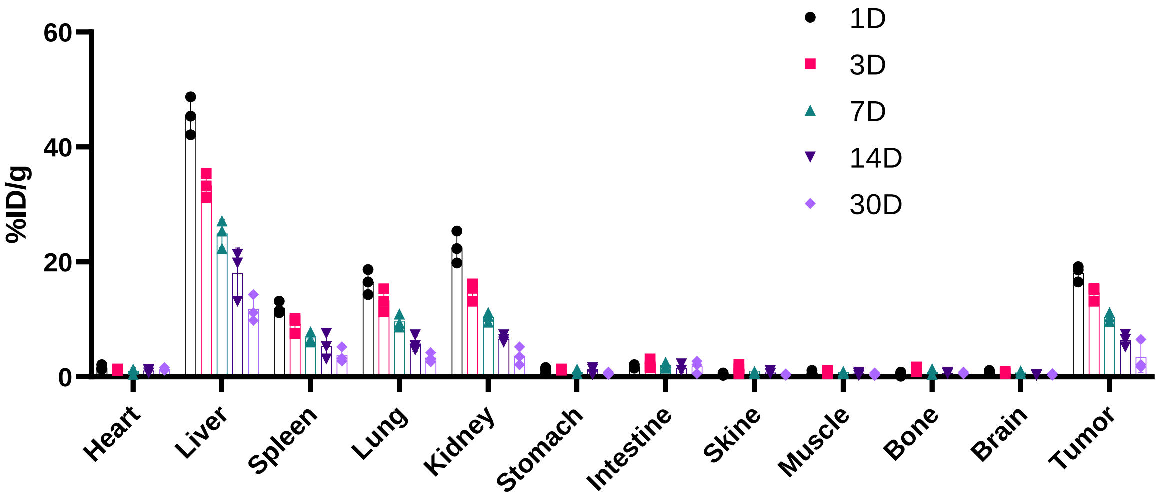

Supplementary Figure 14. Biodistribution of BST/CaO_2 NSs at different times. Data are presented as the mean ± s.d. (n = 3 biologically independent mice).

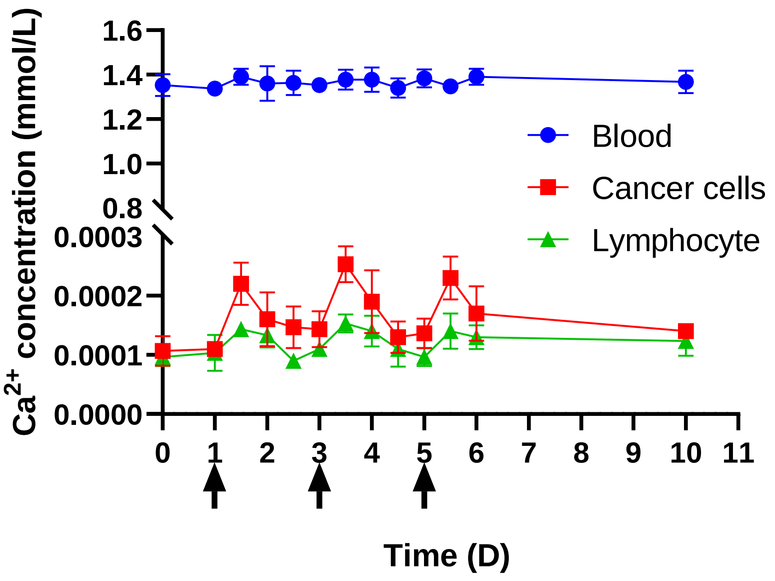

Supplementary Figure 15. The concentrations of Ca^{2+} in blood, tumor cells, and lymphocytes at different times under treatment with BST/CaO_2 NSs. The black arrow represents the three injection times. Data are presented as the mean ± s.d. (n = 3 biologically independent mice).

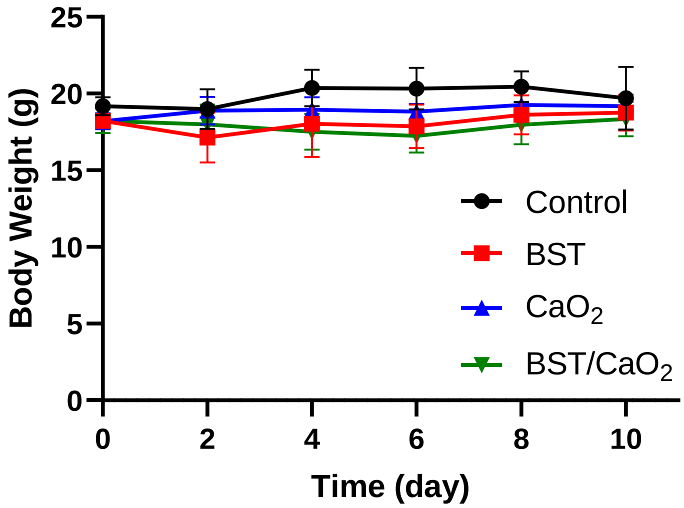

Supplementary Figure 16. The weight curves of tumor-bearing mice under different treatments. Data are presented as the mean ± s.d. (n = 5 biologically independent mice).

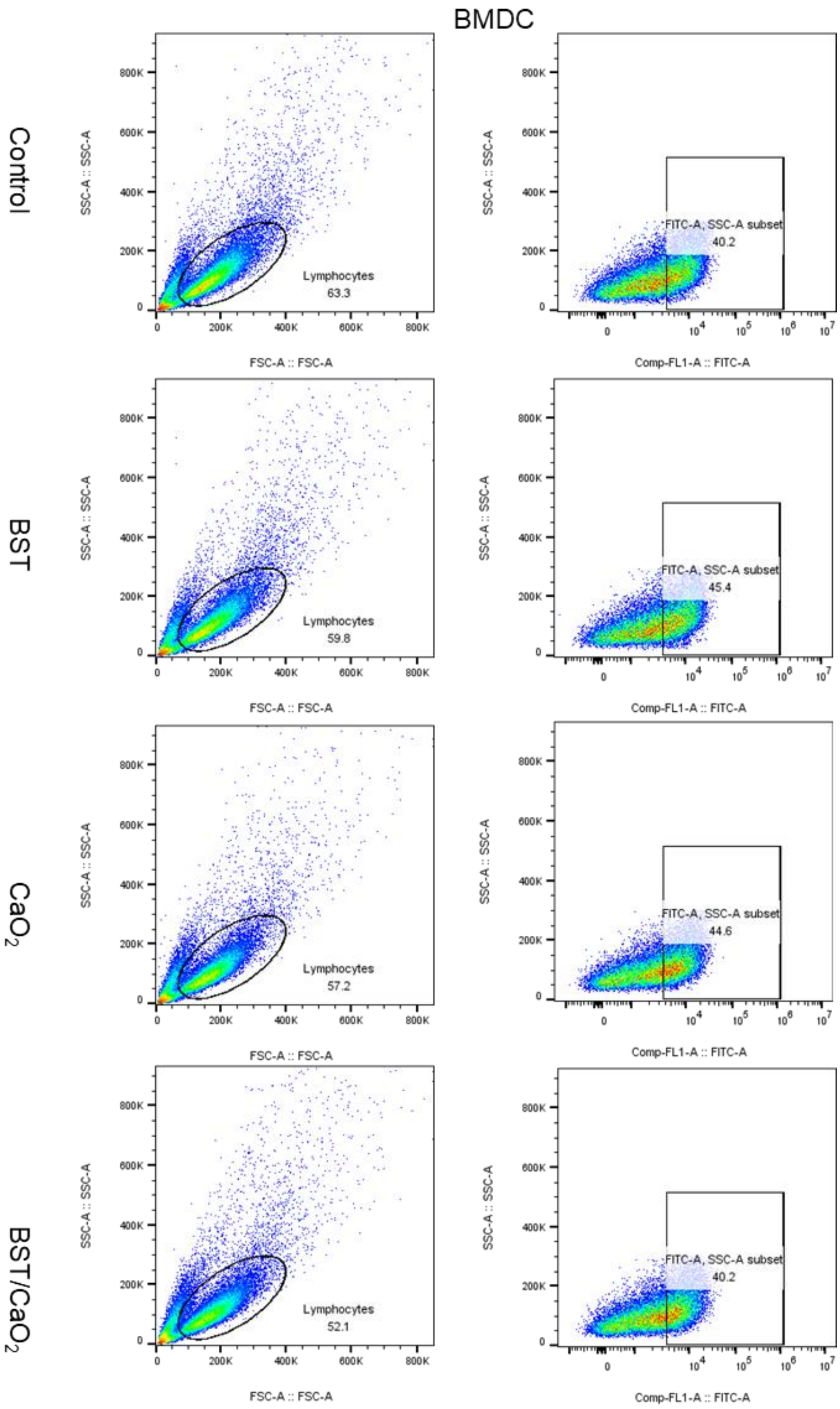

Supplementary Figure 17. Flow cytometry analysis of the percentage of mature DCs ($CD11c^+$ $CD80^+$ $CD86^+$) induced by different treatments. These gating panels correspond to FACS data panels in the Fig. 7g.

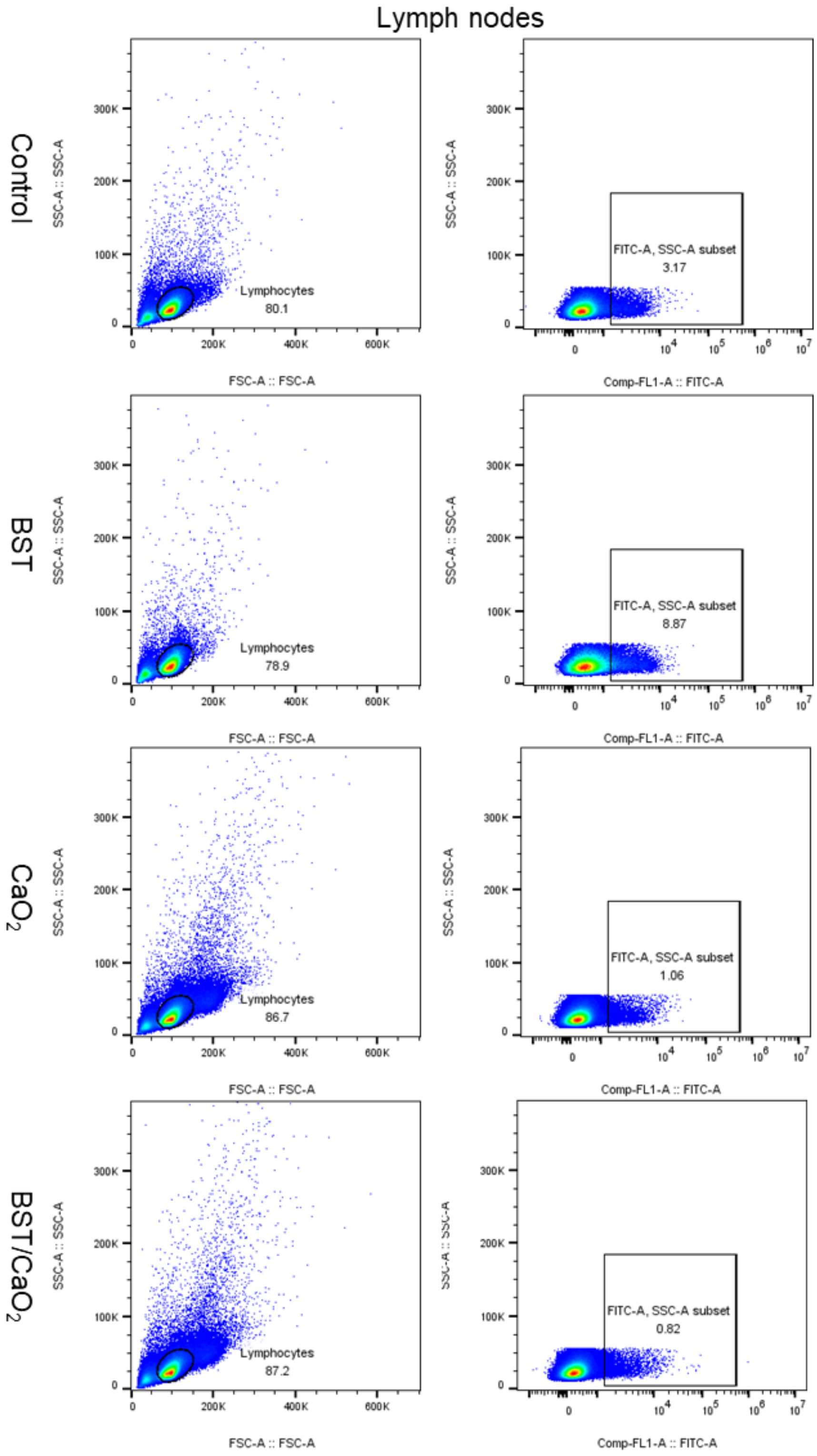

Supplementary Figure 18. Flow cytometry analysis of the percentage of mature DCs in lymph nodes ($CD11c^+$ $CD80^+$ $CD86^+$) induced by different treatments. These gating panels correspond to FACS data panels in the Fig. 7h.

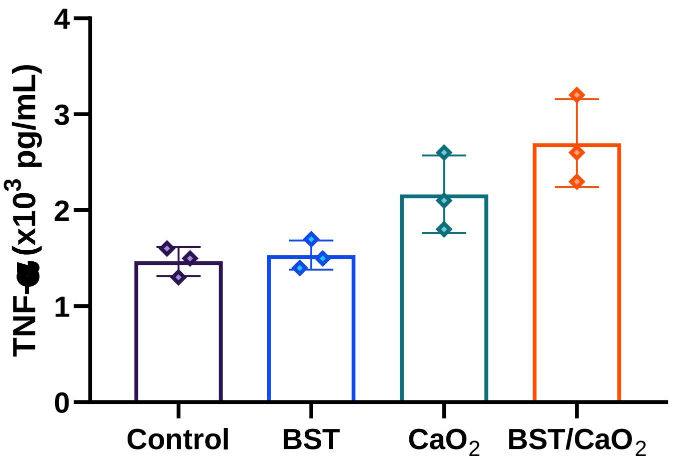

Supplementary Figure 19. Serum levels of TNF-α in tumor-bearing mice under different treatments. Data are presented as the mean ± s.d. (n = 3 biologically independent mice).

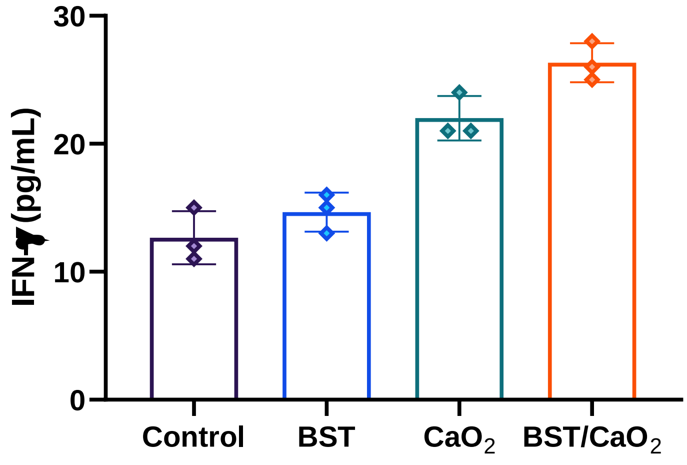

Supplementary Figure 20. Serum levels of IFN-γ in tumor-bearing mice under different treatments. Data are presented as the mean ± s.d. (n = 3 biologically independent mice).

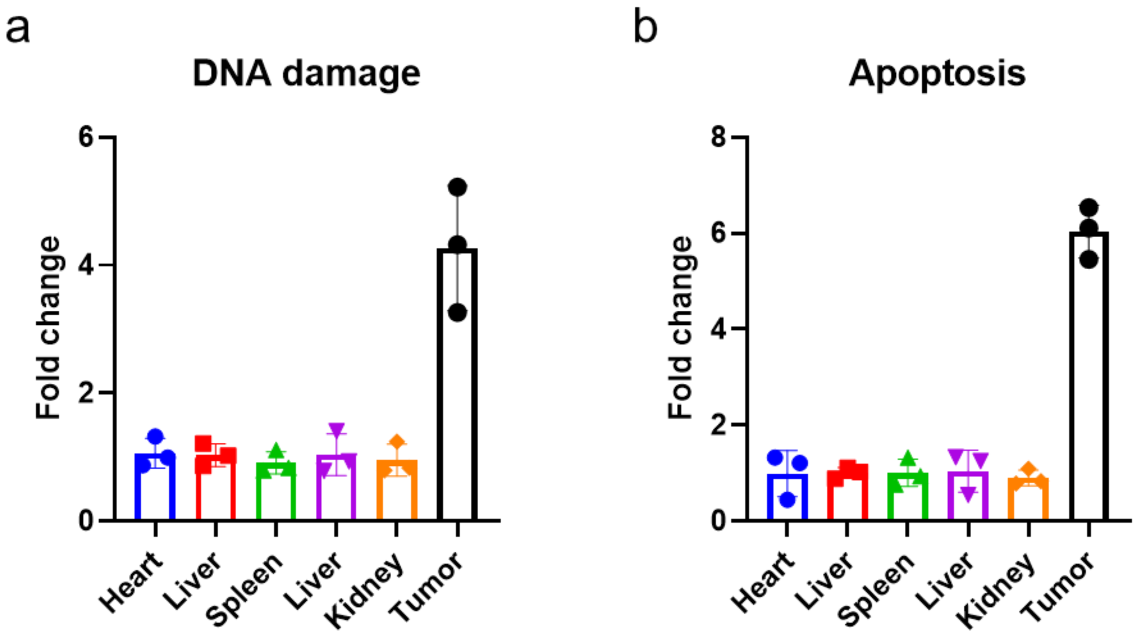

Supplementary Figure 21. Quantitatively measure the apoptosis and DNA damage of organs and tumors after treatment with BST/CaO_2. Data are presented as the mean ± s.d. (n = 3 biologically independent mice).

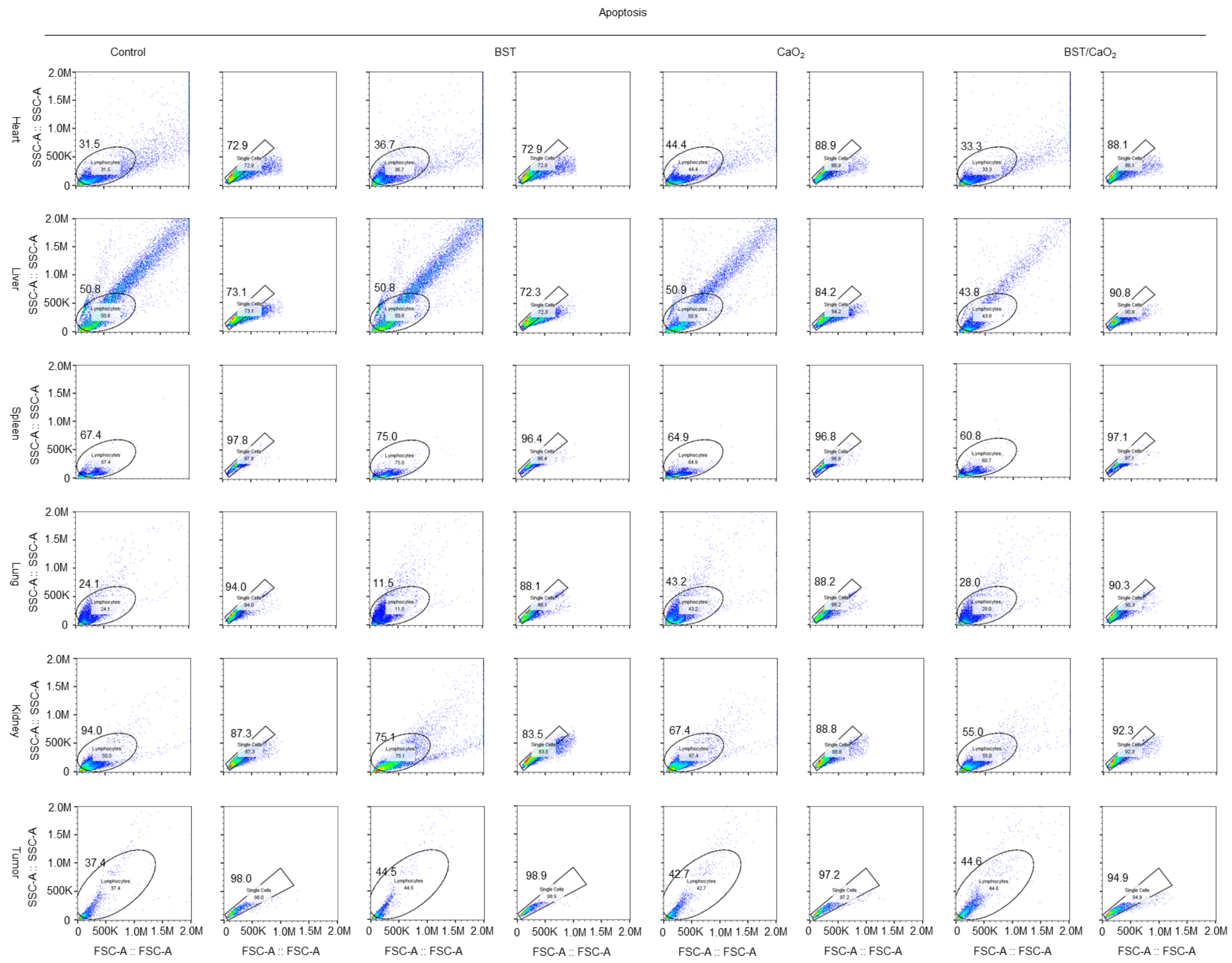

Supplementary Figure 22. Flow cytometry analysis of apoptosis in major organs and tumors induced by different treatments. These gating panels correspond to FACS data panels in the Fig. 9.

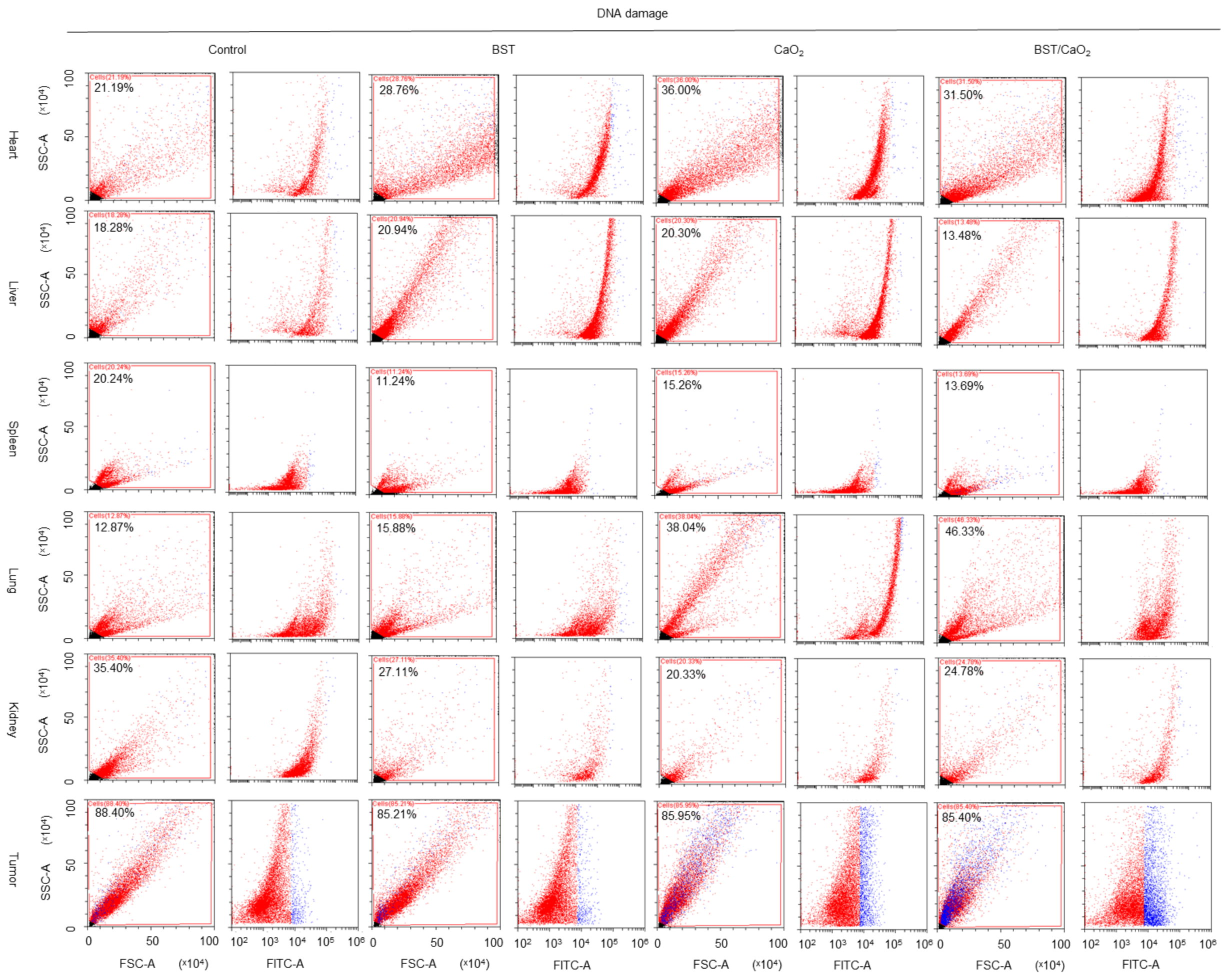

Supplementary Figure 23. Flow cytometry analysis of DNA damage in major organs and tumors induced by different treatments. These gating panels correspond to FACS data panels in the Fig. 9.

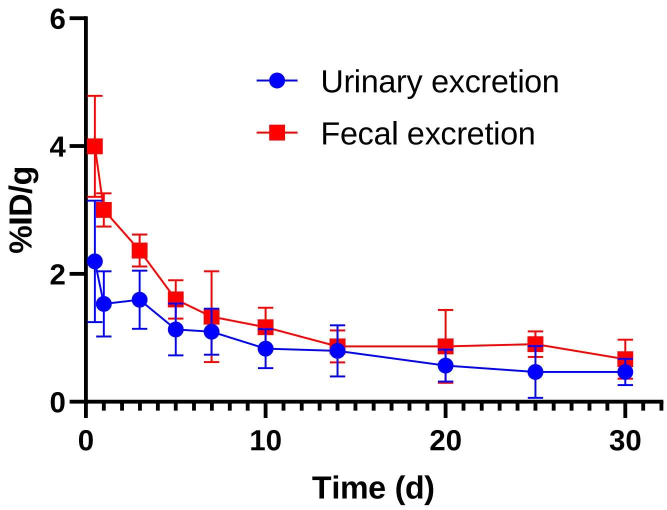

Supplementary Figure 24. Urinary excretion and fecal excretion of BST/CaO_2 NSs at different time points. Data are presented as the mean ± s.d. (n = 3 biologically independent mice).
